# Creative cognition and systems biology on the edge of chaos

**DOI:** 10.3389/fpsyg.2014.01104

**Published:** 2014-09-30

**Authors:** Robert M. Bilder, Kendra S. Knudsen

**Affiliations:** ^1^Department of Psychiatry and Biobehavioral Sciences, David Geffen School of Medicine at UCLA, University of California, Los AngelesLos Angeles, CA, USA; ^2^Semel Institute for Neuroscience and Human Behavior, University of California, Los AngelesLos Angeles, CA, USA; ^3^Department of Psychology, University of California Los AngelesLos Angeles, CA, USA

**Keywords:** creativity, cognition, systems biology, mental illness, schizophrenia, bipolar disorder, dopamine, norepinephrine

Complexity theorists have suggested that production “on the edge of chaos” is important to self-organization and evolutionary change in thermodynamic systems, biology, and economics. We apply this heuristic to cognitive systems and neural network activation states, which can vary from an ordered (predictable) regime, to a chaotic (unpredictable) regime. Evolutionary cytoarchitectonic theory specifies complementary anatomical systems governing stability and flexibility. Psychopathology is associated with shifts in the regulation of stability and flexibility, and may yield both increased redundancy and increased entropy *within* the same individual. We suggest this fits existing literature showing: (a) examples of exceptional creativity in individuals with mental illness, without an overall increase in creative achievement associated with “madness”; (b) increases in creative achievement among relatives of people with mental illness, or people with milder syndromes, for whom increased flexibility or stability is less disabling; and (c) effects of pharmacological manipulations, suggesting an inverted-U function resembling the Yerkes-Dodson Law, possibly linked to tonic and phasic dopaminergic transmission.

## Defining creativity on the edge of chaos for cognitive and biological research

Creative works may be defined as combining novelty (or originality) and utility (or effectiveness, or value) within the domain from which they emerge (Runco and Jaeger, 2012). We relate this to the ideas of Stuart Kauffman, who highlighted the importance of production “on the edge of chaos” to yield valuable change in self-organizing systems spanning thermodynamic, economic, and biochemical systems (such as those involved in the origins of life) (Kauffman, 1993, 1995). Kauffman argues that these systems can be characterized by the predictability of their components. Considering elements within complex systems, some are “orderly” (predictable, redundant) while others are more “chaotic” (unpredictable, entropic). Kauffman sees new and useful developments as emerging “on the edge of chaos,” the boundary between ordered and chaotic regimes.

The Edge of Chaos theory can be applied to cognitive processes and brain activation states important for creative cognition. Considering the diversity of possible cognitive states, we can differentiate the highly predictable and orderly from the unpredictable and chaotic. In more chaotic regimes, network states are more disconnected from those in the ordered regime. But “at the edge of chaos,” the states are maximally novel while still connected to states in the ordered regime, and thus are most likely to manifest the combination of novelty and utility that is the hallmark of creativity. A similar conceptual approach was used to distinguish “rigidity,” “chaos,” and “integration” to characterize semantic network states in people with Asperger's syndrome, schizophrenia, and healthy semantic processing, respectively (see Faust and Kenett, 2014 for good graphical models; see also Siegel, 2010; Kaufman, 2014 for additional examples).

The theory of evolutionary cytoarchitectonic trends may provide an anatomic and neuropsychopharmacologic substrate for these cognitive dimensions, with complementary systems that increase the stability or flexibility of cognitive states via the archicortical and paleocortical trends, respectively (Christensen and Bilder, 2000; Bilder, 2012). Local cortical networks employ the complementary actions of tonic and phasic dopamine signaling, which putatively mediate stability and flexibility, respectively (Bilder et al., 2004); similarly, D1- and D2-like dopamine transmission may mediate persistence or updating within cell assemblies (Durstewitz and Seamans, 2002). Others have emphasized noradrenergic mechanisms to achieve similar network dynamics (McClure et al., 2006). We believe this view advances conceptualization of the role of DA in creative cognition beyond models in which DA was suggested to have a unitary function by facilitating “working memory” or “mental associations” (Flaherty, 2011), or “flexibility” as manifest in higher-order personality traits (DeYoung, 2006).

This theory may help provide a neural systems basis for the theory that creativity results from Blind Variation and Selective Retention (BVSR) (Campbell, 1960; Simonton, 2011a,b). Assuming that blind variation relies on “flexibility” while selective retention relies on “stability,” then our theory has multiple implications: (a) the neural system basis for “sighted” vs. “blind” variations is synaptic facilitation governing the likelihood that a given network activation state will be stabilized and more likely to re-occur; (b) the “unit of analysis” upon which blind variation operates was first stated as a “thought trial” (Campbell, 1960); Simonton refined this to be the “ideational variant”; we suggest that BV operates on the “perception-action cycle,” with a frequency of about 3 Hz, approximately how long it takes to compare a stimulus to expectations; and (c) we distinguish systematic from stochastic variation, with the systematic approach engaging cognitive control (task positive networks), while the stochastic approach disengages cognitive control and engages default mode networks.

## The edge of chaos, creativity, and mental health

How do these ideas relate to the theme of this issue: *creativity and madness*? We suggest the link between creativity and mental illness can benefit from considering the stability and flexibility of cognitive states. For example, Martin Paulus and colleagues used a two-choice guessing paradigm to calculate the entropy of sequential responses made by people with schizophrenia (Paulus et al., 1999). In this paradigm, people “guess” which of two alternatives (e.g., “left” or “right”) will occur next, when in fact the order of outcomes is random. We can analyze the sequence of guesses and quantify their entropy (unpredictability). Healthy people tend to have a Gaussian distribution of sequential guesses, with most responses at intermediate levels of entropy, and fewer very redundant or very entropic responses. In contrast, people with schizophrenia tended to show both more redundant (predictable) and more entropic (unpredictable) responses; longer periods of predictable behavior were interrupted by very unpredictable behavior. We showed that the entropy of responses is linked to dopamine (DA) dynamics; specifically we found that DA “tone” (the balance of agonism to antagonism) had an “inverted-U” association with predictability of responses (Bilder et al., 1992). These examples highlight that the *balance* of stability and flexibility is critical to maintain optimal, healthy function.

The Edge of Chaos theory is also compatible with the neural network frameworks that Ralph Hoffman, Stephen Grossberg, and their colleagues have used to describe schizophrenia (Hoffman and Dobscha, 1989; Grossberg, 1999, 2000; McGlashan and Hoffman, 2000). Either widespread dysconnectivity or impairment of dopamine dynamics governing the stability and flexibility of neural network activation states can lead a network to get “stuck” in a local energy minimum (thus explaining abnormal predictability), but when the activation state does shift, the new state is likely to be more “distant” and less “connected” to the preceding state (thus explaining abnormal unpredictability).

We believe this model conforms with the inverted-U model relating creativity with psychopathology traits and genetic risk for psychosis (Richards et al., 1988) and with existing literature showing examples of exceptional creativity in individuals with mental illness, even though the aggregated results tend to show no overall increase in creative achievement for those with severe mental illness (Rothenberg, 1983; Eisenman, 1990; Abraham et al., 2007; Jaracz et al., 2012). Consistent reports suggest that healthy relatives of those with schizophrenia, and those with schizotypal traits, may have elevated creativity; we suggest that these individuals may have a tendency to greater network flexibility, but additional traits help protect them from developing schizophrenia (Schuldberg, 2000; Kinney et al., 2001; Karimi et al., 2007; Abraham and Windmann, 2008; Batey and Furnham, 2008; Nelson and Rawlings, 2010). Consistent with this are Kinney et al. (2001) results showing higher levels of creativity in people with intermediate levels of schizotypal or schizoid traits, relative to those with lower levels of these traits *and* relative to those with overt schizophrenia.

Evidence about bipolar disorder is less clear, but empirical studies show similar inverted-U distributions: a milder degree of mood disorders, bipolar temperaments, or genetic liability (without full-blown bipolar disorder) may be linked to increased creative achievement, but more severe illness is not. For example, children with pediatric bipolar disorder perform worse on tests of set-shifting or cognitive flexibility (Gorrindo et al., 2005; Dickstein et al., 2007), and while adults with bipolar disorder may have increased inventiveness, scores are lower during depressive episodes (Rybakowski and Klonowska, 2011). Richards et al. (1988) also reported that participants with cyclothymia, and healthy first-degree relatives of patients with bipolar disorder, had higher lifetime creativity scores compared to patients with bipolar disorder and healthy controls with no family history of major affective disorder or schizophrenia. Similarly, researchers found that hypomanic (Furnham et al., 2008) or hyperthymic traits in healthy individuals (Shapiro and Weisberg, 1999) were significantly correlated with measures of creativity. Overall, the data suggest that hereditary risk (without severe impairment), and/or moderate subclinical variations of bipolar disorder or schizophrenia may be associated with enhanced creative achievement, while the more severe forms of these syndromes are associated with impairments of creativity, paralleling impairments in other cognitive processes.

With respect to the evolutionary advantages, Kauffman wrote:

“If it proves true that selection tunes genomic systems to the edge of chaos, then evolution is persistently exploring networks constrained to this fascinating ensemble of dynamical systems (Kauffman, 1993, p. 522).”

We suggest that the underlying genomic systems, and their systems biology correlates at the level of neural network activation states, are tuned to the edge of chaos, helping explain both the observed associations *and dissociations* of creativity with mental illness.

These assumptions are consistent with the hypothesis that neural network dynamics associated with cognitive flexibility are linked to creative achievement because these dynamics generate activation states that are novel with respect to population averages, and favor performance on metrics that are tuned to *divergent thinking* (problem-solving processes that involve exploring multiple alternative solutions). But if the dynamics proceed too far in the direction of flexibility, entropy, and unpredictability, then cognitive products may be novel and unpredictable, but may be “over the edge of chaos,” and not be perceived as useful, valuable, or effective.

## Implications for mental health and promoting creative achievement

The Edge of Chaos hypothesis may help understand the pathophysiology and treatments of mental illness, and suggest paths to augment creativity. We can assess positive traits of cognitive stability and flexibility (or disadvantageous traits: rigidity and lability), and determine their relations with cellular and neuromodulatory factors. For example, widespread dysconnectivity and DA dysregulation remain candidates in the pathophysiology and treatment of schizophrenia. To the extent that interventions impact these states, monitoring stability/flexibility may be beneficial in determining when treatments are managing chaotic (overly flexible) states but not causing cognitive rigidity. For example, measures of network stability/flexibility could help titrate treatments in schizophrenia to maximize freedom from positive symptoms while minimizing cognitive impairment, or titrate the DA and norepinephrine reuptake inhibition in Attention Deficit/Hyperactivity Disorder (ADHD) to maximize attentional control.

Non-pharmacological treatments might someday be tuned to optimize flexibility and stability. For example, EEG neurofeedback strategies emphasizing cognitive stability or flexibility may enhance creativity (Gruzelier, 2014; Gruzelier et al., 2014a,b; but see also Schaller et al., 2013). Slow cortical potential neurofeedback training may enhance stability and benefit those with ADHD (Monastra, 2008; Studer et al., 2014). Transcranial magnetic stimulation or direct current stimulation may also moderate cortical stability and flexibility (Nitsche et al., 2009). TDCS studies already have shown enhancements of creative thinking (Chi and Snyder, 2011; Metuki et al., 2012; Chrysikou et al., 2013; but see also Ghacibeh et al., 2006).

There are further links of cognitive stability and flexibility to meditation practices referred to as focused attention and open monitoring, respectively (Lutz et al., 2008a,b; Slagter et al., 2011). We are not aware of studies showing enhanced creativity among Buddhist monks, but other studies already have shown a positive impact of meditation practices on divergent thinking (Horan, 2009; Colzato et al., 2012). In the future, assessment of individual differences in baseline stability and flexibility might lead to prescriptive contemplative practices. These approaches already have gained traction in the management of anxiety by broadening of attention using mobile phone applications (Enock et al., 2014), and we anticipate the future will deliver additional tools to manage brain activation states, perhaps combined with personal EEG devices.

In conclusion, we believe that understanding both mental illness and creative cognition from the perspective of neural network dynamics, and specifically the regulation of the stability and flexibility of cortical activation states, helps to clarify the relation between creativity and mental illness. We further believe understanding these cognitive dynamics may have profound implications for both understanding of pathophysiology of mental illness, the development of novel intervention strategies for those who are ill, and the enhancement of creative cognition more broadly across the population.

### Conflict of interest statement

The authors declare that the research was conducted in the absence of any commercial or financial relationships that could be construed as a potential conflict of interest.
